# Risk–benefit trade-offs and precision utilities in phase I-II clinical trials

**DOI:** 10.1177/17407745231214750

**Published:** 2023-12-18

**Authors:** Pavlos Msaouel, Juhee Lee, Peter F Thall

**Affiliations:** 1 Department of Genitourinary Medical Oncology, The University of Texas MD Anderson Cancer Center, Houston, TX, USA; 2 Department of Translational Molecular Pathology, The University of Texas MD Anderson Cancer Center, Houston, TX, USA; 3 David H. Koch Center for Applied Research of Genitourinary Cancers, The University of Texas MD Anderson Cancer Center, Houston, TX, USA; 4 Department of Statistics, University of California Santa Cruz, Santa Cruz, CA, USA; 5 Department of Biostatistics, The University of Texas MD Anderson Cancer Center, Houston, TX, USA

**Keywords:** Covariate-specific utilities, phase I-II trials, personalized medicine, prognostic subgroups, risk–benefit trade-offs, utility functions

## Abstract

**Background:**

Identifying optimal doses in early-phase clinical trials is critically important. Therapies administered at doses that are either unsafe or biologically ineffective are unlikely to be successful in subsequent clinical trials or to obtain regulatory approval. Identifying appropriate doses for new agents is a complex process that involves balancing the risks and benefits of outcomes such as biological efficacy, toxicity, and patient quality of life.

**Purpose:**

While conventional phase I trials rely solely on toxicity to determine doses, phase I-II trials explicitly account for both efficacy and toxicity, which enables them to identify doses that provide the most favorable risk–benefit trade-offs. It is also important to account for patient covariates, since one-size-fits-all treatment decisions are likely to be suboptimal within subgroups determined by prognostic variables or biomarkers. Notably, the selection of estimands can influence our conclusions based on the prognostic subgroup studied. For example, assuming monotonicity of the probability of response, higher treatment doses may yield more pronounced efficacy in favorable prognosis compared to poor prognosis subgroups when the estimand is mean or median survival. Conversely, when the estimand is the 3-month survival probability, higher treatment doses produce more pronounced efficacy in poor prognosis compared to favorable prognosis subgroups.

**Methods and Conclusions:**

Herein, we first describe why it is essential to consider clinical practice when designing a clinical trial and outline a stepwise process for doing this. We then review a precision phase I-II design based on utilities tailored to prognostic subgroups that characterize efficacy–toxicity risk–benefit trade-offs. The design chooses each patient’s dose to optimize their expected utility and allows patients in different prognostic subgroups to have different optimal doses. We illustrate the design with a dose-finding trial of a new therapeutic agent for metastatic clear cell renal cell carcinoma.

## Introduction

Only 10% of new agents that enter phase I clinical testing will be successful in reaching the market, while 68% and 40% of agents will fail during phases II and III, respectively.^1^ Finding the right dose in an early-phase trial is crucial because therapies administered at doses that are unsafe or biologically ineffective are unlikely to be successful in subsequent phases or to achieve regulatory approval. Choosing the right dose or doses for a new therapy requires evaluation of risk–benefit trade-offs for outcomes such as biological efficacy, toxicity, and quality of life.^2–4^ In contrast with traditional phase I trials that determine a dose based solely on toxicity, phase I-II trials explicitly account for both efficacy and toxicity and use these to identify doses that provide the most desirable risk–benefit trade-offs.^5,6^ In recent years, it has become increasingly evident that one-size-fits-all dose optimization methods may be inadequate because a patient’s covariates, such as a laboratory or clinical biomarker, often play important roles in determining what dose is most appropriate. Consequently, dose-finding designs should account for patient heterogeneity in the outcomes and risk–benefit trade-offs they wish to prioritize.

To establish a context for the phase I-II design that we will review, we first describe a multistep process that provides a coherent basis for designing a dose-finding trial with personalized treatment decisions. We consider the way in which a physician may choose an individual patient’s treatment in clinical practice and outline corresponding procedures to construct and implement a dose-finding design. We demonstrate that a precision phase I-II design integrates not only individual patient covariates for modeling dose-outcome relationships, but also subjective risk–benefit trade-offs between desired and adverse outcomes through a utility function for improved dose selection. We also illustrate how a utility function may be constructed to vary among patients based on their specific covariates. We then review a precision phase I-II dose-finding design that uses utility functions tailored to prognostic subgroups, where a utility function quantifies risk–benefit trade-offs between toxicity and efficacy for each subgroup. We illustrate this design with a phase I-II trial for a new agent to treat metastatic clear cell renal cell carcinoma (mccRCC).^4^

## Connecting clinical trial design to clinical practice

While it is a truism that a clinical trial design should reflect real-world clinical practice, it may not be obvious how to construct such a design. To provide a coherent structure, we will describe steps for informed decision-making in medical practice, with corresponding steps for the design and conduct of a dose-finding trial. These are given in Table 1, including a column for clinical practice and a corresponding column for designing and conducting a phase I-II dose-finding trial. For a Bayesian model-based phase I-II design, model specification typically involves selecting a noninformative or weakly informative prior distribution, and model fitting involves iteratively updating the posterior distribution based on the most recently available data. Estimation consists of computing posteriors of quantities of interest, such as probabilities of response and toxicity, and decision criteria may be based on posterior quantities or predicted values as functions of treatment or doses and patient covariates.

**Table 1.** Connecting clinical practice to phase I-II clinical trial design and conduct.

Task	Physician actions	Phase I-II clinical trials
		Trial design
Determine the disease	Diagnose the patient’s disease	Specify the disease and trial entry criteria
Determine key elements	Specify clinical outcomes, patient covariates, and identify possible treatments	Define response, toxicity, and other key outcomes; identify patient prognostic subgroups; and determine doses to be studied
Establish a statistical model	Specify statistical models for outcomes as functions of treatment, dose or schedule, and patient covariates	Specify statistical models for the probabilities of response and toxicity as functions of dose or schedule and patient subgroup
Establish utility functions	Determine utilities as functions of outcomes and patient covariates	Determine utilities as functions of response, toxicity, and subgroup
Identify key estimands	Identify key estimands as clinically interpretable functions of model parameters, including mean utilities	Identify subgroup-specific probabilities of toxicity, response, and subgroup-specific mean utilities as criteria for clinical decision-making during the trial
		Trial conduct
Obtain data	Obtain data from clinical practice databases or published studies	Obtain the most recently updated data from patients treated previously in the trial
Analyze the data	Fit the statistical model to the data	Fit the statistical model to the most recent trial data
Compute estimates	Compute estimates of all relevant estimands, including mean utilities and predicted outcomes, as functions of the patient’s covariates for each potential treatment	Compute estimates of the probabilities of response and toxicity, the acceptable dose set as functions of patient covariates and dose and mean utility for the doses in the set
Make a decision and take action	Make a clinical decision based on the estimates and treat the patient with the chosen treatment	Choose an optimal acceptable dose, or do not treat the patient if no dose is acceptable

The International Council for Harmonization (ICH) of Technical Requirements for Registration of Pharmaceuticals for Human Use recently released the ICH E9(R1) guidance that defines “estimands,” that is, a quantity to be estimated in a statistical analysis, to be used when optimizing risk–benefit assessments, depending on the clinical context.^7–9^ A recent review highlights the need to use estimands that incorporate treatment effects on efficacy, toxicity, and patient-reported quality of life (QOL).^7^ Ideally, estimands should be readily interpretable by practicing physicians and defined as functions of treatment or dose and subgroup as determined by patient covariates. Examples of commonly used estimands include the probability of toxicity or response within a specified time from treatment initiation, probability of survival beyond a specified time, mean or median survival time, and hazard ratio for survival time.^3^ Importantly, the choice of an estimand may have a profound effect on treatment selection, with different estimands giving different treatment effect evaluations across patient subgroups. This will be illustrated below, where we will see that when the estimand is mean or median survival, higher treatment doses can yield much higher efficacy in patient subgroups with favorable prognosis compared with poor prognosis, whereas when the estimand is 3-month survival probability, efficacy is more pronounced in poor-prognosis subgroups.

To choose an optimal treatment, we assign a numerical utility to each outcome, allowing these to vary by patient subgroup. Risk–benefit tradeoffs for a specific subgroup are reflected in the values assigned to combinations of outcomes and subgroups. Examples include U(response, toxicity, subgroup) in a dose-finding trial^4^ or U(PFS, QOL, subgroup) in a randomized comparative trial,^10^ where PFS denotes progression-free survival and QOL denotes quality of life. For example, Lee et al.^11^ provided a decision analysis based on the preference of older patients with advanced breast cancer for treatments associated with lower toxicity and thus better QOL at the cost of lower biological efficacy for extending PFS time. While long PFS time with no toxicity typically has high utility for patients of all ages, the utility assigned to low toxicity but shorter PFS time is greater for older patients compared to younger patients. Consequently, a treatment with high efficacy for extending PFS time but accompanied by high toxicity is less likely to be chosen as optimal for older patients. Such trade-offs are inherently subjective, as reviewed in detail elsewhere,^3,12,13^ but this subjectivity is an important advantage of utility functions, rather than a drawback. If several utility functions corresponding to different risk–benefit trade-offs are under consideration, calculations that identify optimal treatments or doses can be performed using each utility function as a sensitivity analysis that provides physicians and patients with insight into how different utility assignments impact treatment decisions.

## Prognostic and predictive covariates

**Figure 1.**
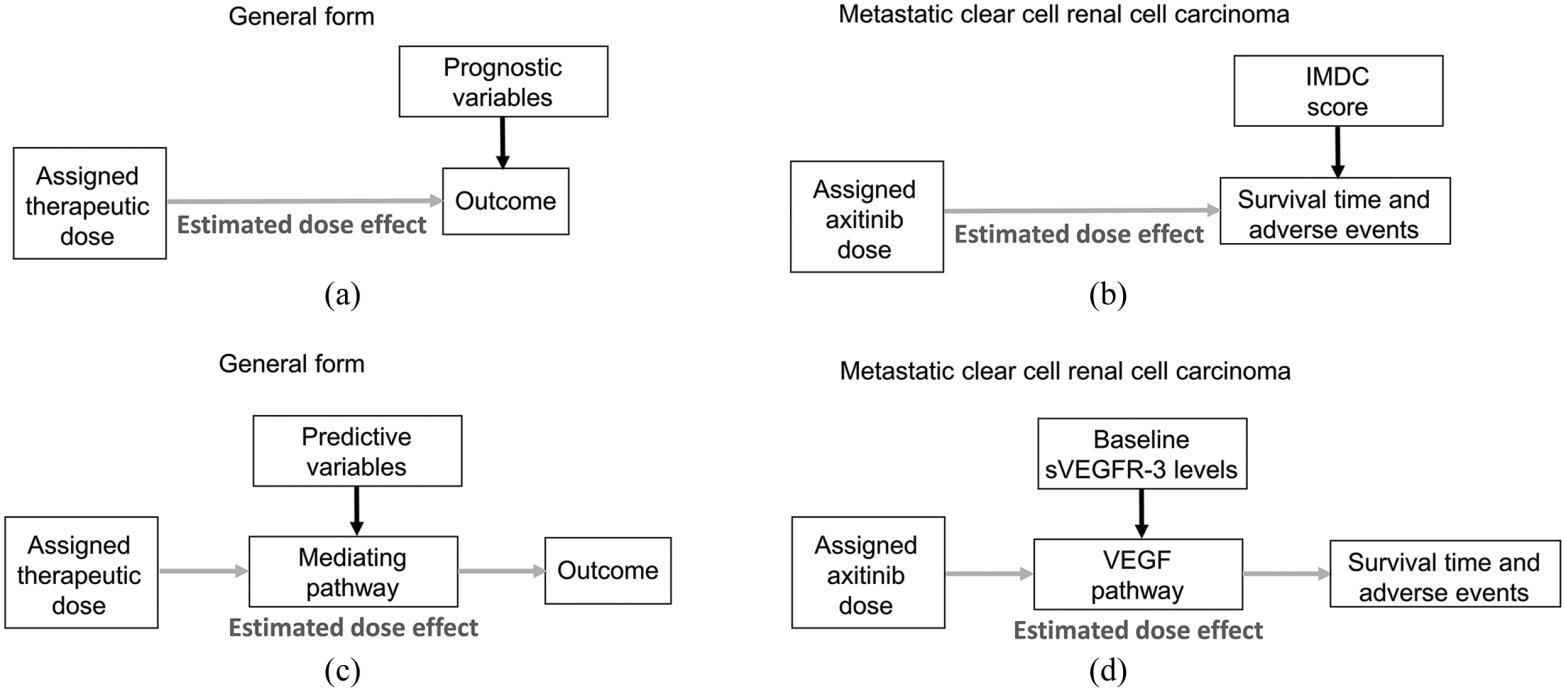
Causal diagrams representing the data-generating processes of prognostic and predictive effects in dose-finding trials. The putative dose effect under investigation is denoted by gray arrows. (a) Prognostic biomarkers are baseline patient variables that directly influence the outcome and not the estimated dose effect. Thus, the estimated dose effect parameters such as log_e_ odds are assumed to be stable for all patients. (b) Corresponding clinical scenario whereby the International Metastatic Renal Cell Carcinoma Database Consortium (IMDC) score is an established prognostic score that directly influences survival time and adverse events in mccRCC. The assigned dose of the drug axitinib may also impact overall survival. (c) Predictive biomarkers are baseline patient variables that influence the estimated dose effect through their effect on the mediating pathway that transmits the effect of the assigned dose on the outcomes of interest. (d) Corresponding clinical scenario whereby baseline levels of soluble vascular endothelial growth factor receptor 3 (sVEGFR-3) influence the vascular endothelial growth factor (VEGF) pathway that mediates the dose effect of the VEGF inhibitor axitinib on survival time and adverse events.

The magnitude and direction of estimated treatment effects on clinical outcomes are often influenced by baseline patient covariates, also referred to as “moderator variables.”^14,15^ As reviewed extensively,^15^ causal diagrams can be used to distinguish between two different types of moderator effects based on underlying data-generating processes (Figure 1). The first type of moderator effect (Figure 1(a)) is commonly known as a “prognostic effect,” a term we adopt here. It has also been described in the literature as “main effect,”“additive effect,”“risk magnification,”“risk modeling,” or “effect measure modification.”^3,14–19^ As an example in mccRCC, the International Metastatic Renal Cell Carcinoma Database Consortium (IMDC) prognostic score is used by organizations such as the National Comprehensive Cancer Network to guide therapeutic decisions. This score is calculated by combining laboratory and clinical variables such as anemia, thrombocytosis, neutrophilia, hypercalcemia, performance status, and time from diagnosis to treatment.^20,21^ Based on their IMDC scores, patients with mccRCC can be categorized into favorable-, intermediate-, or poor-risk prognostic subgroups.^20,22,23^ IMDC score is an established prognostic factor influencing survival outcomes for drugs such as axitinib that target the vascular endothelial growth factor (VEGF) pathway.^20^ Patients in the poor-risk IMDC subgroup are at higher risk of experiencing adverse events such as fatigue and weight loss compared to patients with favorable risk. These adverse events can contribute to a shorter survival time for patients with poor-risk IMDC disease regardless of the treatment they receive (Figure 1(b)).

The second type of moderator effect, which we call a “predictive effect,” occurs due to a biological interaction between a covariate and a particular treatment via a mediating pathway (Figure 1(c)). A covariate that is predictive for one agent may not be predictive for another. Other names used in the literature include “treatment interaction,”“multiplicative effect,”“biologic interaction,”“effect modeling,” and “biological treatment effect modification.”^3,14–19^ A clinical example in mccRCC is the use of baseline soluble vascular endothelial growth factor receptor 3 (sVEGFR-3) level as a predictive variable that influences the mediating VEGF pathway targeted by axitinib and thus modifies the effect of axitinib on clinical outcomes such as survival time and adverse events (Figure 1(d)).^24^ In clinical data, predictive effects are typically weaker and more challenging to identify than prognostic effects.^3,15,18,25,26^ Due to the challenges in modeling predictive effects, many statistical models used in drug development do not include predictive covariate effects. However, prognostic covariates, which are more likely to have consistent and additive effects on outcomes, are often included in regression models to improve the precision of statistical estimates and outcome predictions.^14,16,27–31^ While we focus here on dose-finding based on risk–benefit trade-offs that account for prognostic covariates, our framework can easily be extended to include predictive covariates.

## Prognostic influence on dose finding with time-to-event outcomes

Although, for practical reasons, most phase I-II designs use early efficacy endpoints such as objective response evaluated within 30 days, modern dose-finding designs are beginning to incorporate more long-term clinical outcomes, such as overall survival or PFS time.^32,33^ Toxicity can also be defined as a time-to-event variable monitored continuously over a prespecified follow-up period.^2,4,6,33^ The effect of patient prognosis on a time-to-event outcome may manifest differently based on the estimand employed to characterize a treatment or dose effect, leading to potentially conflicting treatment evaluations.^3,13,34^

As a simple illustration, we assume a proportional hazards model with an exponential distribution for the baseline hazard function in the numerical example below.^35–37^ However, the same phenomenon is observed for models such as a Weibull, gamma, or other distribution with a hazard function that may increase or decrease over time.^34^ Under the assumed model, the hazard (rate), h is constant over time and changes with dose and patient prognostic subgroup. The following examples consider two doses, d = 1 and d = 2, and two prognostic subgroups, s = fav (favorable) and s = poor, resulting in four hazards, h(1, fav), h(2, fav), h(1, poor), and h(2, poor), and two hazard ratios (HRs) comparing dose 2 to dose 1 for each subgroup: HR(fav) = h(2, fav)/h(1, fav) for favorable risk scores and HR(poor) = h(2, poor)/h(1, poor) for poor risk scores.

Suppose that a phase I-II dose-finding trial of a new investigational drug as salvage therapy for mccRCC has been completed, and one wishes to choose between doses 1 and 2. Denote the hazard of death by h_D_(d,s) for dose d and subgroup s, so the HR comparing the two doses in each subgroup is

(1) $${\mathrm{HR}}_{D}(s){=h}_{D}(2,s){/h}_{D}(1,s)$$

**Figure 2.**
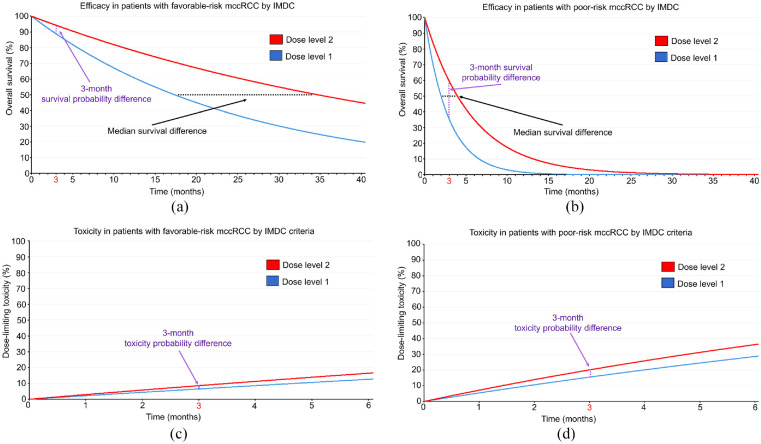
Time-to-event curves assuming an exponential distribution for patients with mccRCC treated with two different dose levels of an investigational therapy in a phase I-II trial. The black dotted lines correspond to median survival difference, whereas the purple dotted lines correspond to the survival probability difference at 3 months. The HR for the efficacy outcome is assumed to be 0.5 favoring dose level 2, and 0.75 for the DLT outcome favoring dose level 1, irrespective of IMDC prognostic risk classification. (a) For the efficacy outcome of OS in patients with favorable-risk IMDC, the survival probability difference at 3 months is 5.5% favoring dose level 2, whereas the median survival difference is 17.3 months favoring dose level 2. (b) For the efficacy outcome of OS in patients with IMDC poor risk, the survival probability difference at 3 months is 24.1% favoring dose level 2, whereas the median survival difference is 2 months favoring dose level 2. (c) For the DLT outcome in patients with IMDC favorable risk, the toxicity probability difference at 3 months is 2.1% favoring dose level 1. (d) For the DLT outcome in patients with IMDC poor risk, the toxicity probability difference at 3 months is 4.6% favoring dose level 1.

Suppose that dose 2 yields greater benefit than dose 1 in overall survival (OS) time, with estimated HR_D_(fav) = HR_D_(poor) = 0.5 in both IMDC risk groups. For time to toxicity, suppose that dose 2 is more likely to produce dose-limiting toxicities (DLTs) than dose 1, with an estimated dose 2 to dose 1 HR_T_(fav) = HR_T_(poor) = 1/0.75 = 1.33 that is stable across IMDC risk groups. Figure 2 shows the time-to-event curves for efficacy and toxicity stratified by IMDC favorable- or poor-risk prognostic subgroups. Under the exponential distribution, HR_D_ = 0.5 implies that the median survival of patients treated with dose 2 is double that of patients treated with dose 1. Accordingly, if patients with favorable-risk IMDC prognosis have a median OS of 17.3 months when treated with dose 1, then the median OS with dose 2 is 34.6 months for a median OS difference of 34.6 – 17.3 = 17.3 months. Under an exponential distribution, mean = median/log_e_(2), and therefore, the difference in mean OS between dose 2 and dose 1 is 49.92 – 24.96 = 24.96 months for favorable-risk patients. Patients with poor-risk IMDC prognosis have a median OS of 4 months with dose 2 and 2 months with dose 1 for a dose 2 versus dose 1 median OS difference of 4 – 2 = 2 months and mean OS difference of 5.77 – 2.89 = 2.88 months. Therefore, in terms of mean or median survival time, the benefit of dose 2 over dose 1 is far greater for patients with IMDC favorable risk than for patients with poor risk (Figure 2(a) and (b)).

A different conclusion is reached, however, if one focuses on a different estimand, namely survival probabilities beyond a given milestone time point, defined by the *survival probability difference* (SPD) of dose 2 versus dose 1 in subgroup s at 3 months

(2) $$\mathrm{SPD}\left(3,s\right)=P\left(T_{D}>3|d=2,s\right)-P\left(T_{D}>3|d=1,s\right)$$

where T_D_ denotes survival time (Figure 2(a) and (b)). For each dose (d = 1 or 2) and IMDC subgroup (s = fav or poor), let h_D_(d,s) denote the hazard of death for that dose and subgroup. Under an exponential distribution, the survival probability of patients treated with dose d in IMDC subgroup s is P(T_D_ > t—d, s) = exp{−h_D_(d,s) t}, where t is a milestone time point of interest, such as t = 3 months. The exponential distribution has h = 1/mean, and the 3-month SPD for patients with IMDC favorable risk is

SPD(3,fav)=P(T>3|d=2,fav)-P(T>3|d=1,fav)=.942−.887=.055

For patients with IMDC poor risk, the SPD is

SPD(3,poor)=P(T>3|d=2,poor)-P(T>3|d=1,poor)=.595-.354=.241

Thus, in terms of 3-month survival probabilities, the benefit of dose 2 over dose 1 is much larger for patients with IMDC poor risk than for patients with favorable risk (Figure 2(a) and (b)).

This numerical example shows that, if one wishes to account for a patient’s subgroup when comparing doses in order to choose a best personalized dose, how “best” is defined depends on the estimand used to evaluate the doses. Different estimands can give very different answers, even for a specific endpoint such as survival time. Thus, one must decide, for example, whether mean survival time or survival probability beyond 3 months is what matters most in evaluating treatments.

Time to toxicity, Y_T_, also depends on the particular shapes of the survival distribution curves being compared, which may change with patient prognosis (Figure 2(c) and (d)). Suppose that dose 2 has a higher DLT hazard at any time compared to dose 1 with HR_T_(fav) = HR_T_(poor) = 1/0.75 = 1.33 that is stable across IMDC risk groups. Because many modern phase I-II trials in oncology, including mccRCC trials, use 3 months from treatment initiation as the evaluation window for DLTs, we will focus on the *toxicity probability difference* (TPD) of dose 2 versus dose 1 in subgroup s at 3 months

(3) $$\mathrm{TPD}\left(3,s\right)=P\left(Y_{T}<3|\mathrm{dose}=2,s\right)-P\left(Y_{T}<3|\mathrm{dose}=1,s\right)$$

where Y_T_ denotes time to DLT (Figure 2(c) and (d)). Assuming monotonicity, higher doses increase both efficacy and toxicity. For each dose (d = 1 or 2) and IMDC subgroup (s = favorable or poor), let h_T_(d,s) denote the hazard of DLT for that dose and subgroup. Under an exponential distribution, the toxicity probability of patients treated with dose d in IMDC subgroup s is P(Y_T_ < t| d, s) = 1 − exp{−h_T_(d,s) t}, where t is a milestone time point of interest, such as t = 3 months. If the toxicity probability at 3 months for dose 2 in the favorable-risk group is P(Y_T_ < 3| dose = 2, fav) = .086, then h_T_(2, fav) = .03. Assuming HR_T_(fav) = h_T_(2, fav)/h_T_(1, fav) = 1/0.75 = 1.33, we obtain h_T_(1, fav) = 0.0225, which gives P(Y_T_ < 3| dose = 1, fav) = .065. Therefore, the 3-month TPD for patients with IMDC favorable risk is

$$\mathrm{TPD}\left(3,\mathrm{fav}\right)=P\left(Y_{T}<3|d=2,\mathrm{fav}\right)-P\left(Y_{T}<3|d=1,\mathrm{fav}\right)=.086-.065=.021$$

For IMDC poor risk, if the toxicity probability at 3 months for dose 2 is P(Y_T_ < 3| d = 2, fav) = .201, then we can use the same approach to estimate h_T_(2, poor) = .075 and h_T_(1, poor) = .05625 because HR_T_(poor) = h_T_(2, poor)/h_T_(1, poor) = 1/0.75 = 1.33. This allows us to estimate P(Y_T_ < 3| dose = 1, poor) =.155. Accordingly, the 3-month TPD for patients with IMDC poor risk is

$$\mathrm{TPD}\left(3,\mathrm{poor}\right)=P\left(Y_{T}<3|d=2,\mathrm{poor}\right)-P\left(Y_{T}<3|d=1,\mathrm{poor}\right)=.201-.155=.046$$

This shows that, in terms of 3-month DLT probabilities, the benefit of dose 1 over dose 2 is larger for patients with IMDC poor-risk mccRCC (Figure 2(c) and (d)).

**Figure 3.**
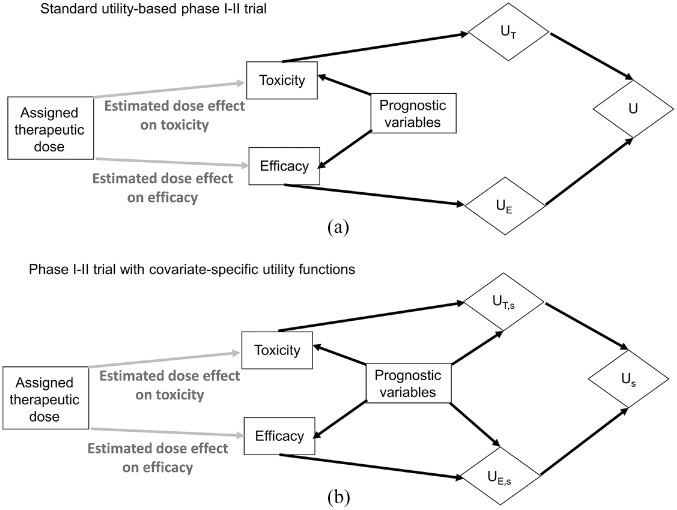
Causal diagrams representing the data-generating processes and corresponding utility nodes (diamonds) in phase I-II dose-finding trials. The putative dose effect under investigation is denoted by gray arrows. (a) Standard utility-based phase I-II trials assign numerical utilities U_T_ to toxicity outcomes and U_E_ to efficacy outcomes. Dose decisions are obtained by calculating the total utility U = U_T_ + U_E_. (b) Phase I-II trials may also use covariate-specific utility functions that are modified based on each patient’s prognostic subgroup, g, to obtain subgroup-specific numerical utilities U_T,s_ for toxicity outcomes and U_E,s_ for efficacy outcomes. Dose decisions are obtained for each prognostic subgroup g by calculating the total subgroup-specific utility U_s_ = U_T,s_ + U_E,s_.

Another key point is that the prioritization of the two quantities, 3-month SPD and 3-month TPD, over more long-term efficacy and toxicity outcomes is itself a decision often done for convenience in phase I-II designs that will impact dose finding. In such cases, patients with poor prognosis may be more likely to obtain early efficacy benefit (Figure 2(a) and (b)), and thus, the design will tend to prioritize doses with higher efficacy in the poor-risk population, at the cost of potentially higher toxicity. Researchers should be aware of this implicit assumption to ensure that risk–benefit trade-offs are properly accounted for during phase I-II dose-finding tailored to prognostic subgroups. This provides a conceptual framework for constructing utility functions that will quantify the risk–benefit trade-offs based on efficacy and toxicity differences between prognostic subgroups (Table 2). A simple view of a utility is that it reduces two outcomes, such as response and toxicity, to one number U(response, toxicity) that quantifies the desirability of each pair of values. The use of different utility functions to prioritize mean/median survival differences or milestone SPD values in the context of mccRCC has been reviewed elsewhere.^3^ Figure 3(a) uses a causal diagram to illustrate a utility function that is influenced by a toxicity and efficacy outcome of interest. Let U_T_(toxicity) and U_E_(efficacy) represent numerical utilities assigned to each toxicity and efficacy outcome. The final dose decision is based on a total utility, which may be defined as the sum

(4) $$U\left(\mathrm{toxicity},\mathrm{efficacy}\right)=U_{T}\left(\mathrm{toxicity}\right)+U_{E}\left(\mathrm{efficacy}\right)$$

**Table 2.** Patient-specific survival outcomes for two doses of a hypothetical experimental therapy for mccRCC with an OS (efficacy time-to-event outcome) HR 0.5 favoring dose 2 over dose 1 and dose-limiting toxicity (time-to-toxicity outcome) HR 1/0.75 = 1.33 favoring dose 1 over dose 2 for IMDC prognostic subgroups.

	Scenario A		Scenario B		Remarks
IMDC prognostic subgroup	Favorable risk		Poor risk		Scenarios A and B differ only in IMDC prognostic status
Median OS (months)	Dose 1 17.3	Dose 2 34.6	Dose 1 2.0	Dose 2 4.0	Median and mean survival differences are larger for IMDC favorable risk compared with IMDC poor risk.
Mean OS (months)	24.95	49.92	2.89	5.77	
Survival probability at 3 months	88.7%	94.2%	35.4%	59.5%	The survival probability difference at 3 months is larger for IMDC poor risk compared with IMDC favorable risk.
Toxicity probability at 3 months	6.5%	8.6%	15.5%	20.1%	The toxicity probability difference at 3 months is larger for IMDC poor risk compared with IMDC favorable risk.

mccRCC: metastatic clear cell renal cell carcinoma; OS: overall survival; HR: hazard ratios; IMDC: International Metastatic Renal Cell Carcinoma Database Consortium.

if U_T_(toxicity) and U_E_(efficacy) are scaled to the same domain. Depending on the setting, U(toxicity, efficacy) may instead be a product of U_T_· U_E_, if U_T_ is scaled to the interval [0, 1], with greater toxicity given smaller U_T_. Patient prognostic factors affect 3-month DLT probabilities and 3-month survival probabilities. Consequently, the distribution and estimates of U(toxicity, efficacy) vary with subgroups and doses, which enables one to optimize treatment selection based on patient characteristics and tailor treatment selections for individual patients. This framework can be generalized to include additional outcome variables, such as patient-reported QOL, which also may be assigned numerical utilities to include when making dose decisions.

## Covariate-specific utility functions

Prognostic variables not only influence the estimation of unknown parameters and prediction of clinical outcomes (Figure 3(a)), but also can change how numerical utilities are assigned to each outcome (Figure 3(b)). In the mccRCC example, patients with IMDC poor risk typically are more willing to accept a higher risk of toxicity to curtail their highly aggressive disease. Conversely, the relatively indolent disease status in patients with IMDC favorable risk makes toxicity less acceptable, particularly since these patients are expected to live much longer. Thus, prognostic subgroups, s, can directly influence the assigned numerical utilities for toxicity U_T,s_ and efficacy U_E,s_. A total utility U_s_(toxicity, efficacy) = U_T,s_ (toxicity) + U_E,s_(efficacy) is tailored to each patient’s prognostic subgroup, for example, IMDC score in mccRCC. Such covariate-specific utility functions have recently been used in phase I-II dose-finding and in randomized phase II trials that have superior properties compared to simpler designs that do not tailor decisions for each prognostic subgroup.^4,10^

In contrast with the mccRCC setting, for other diseases such as certain metastatic breast cancer subtypes, clinicians and patients may prefer treatments with lower toxicity risk for poorer prognostic subgroups, for example, older patients who typically have more comorbidities and shorter expected survival than younger patients. Such covariate-specific utility functions were integrated with data from a phase 3 randomized controlled trial (RCT) using a flexible multivariate Bayesian nonparametric regression model to inform the selection of letrozole alone versus the combination of letrozole plus bevacizumab as first-line therapy for hormone receptor–positive advanced breast cancer.^11^ The utility functions were defined to vary with age because older patients with this type of breast cancer typically are less willing to accept more intensive therapies that can prolong survival outcomes at the cost of severe toxicities. The practical requirements of these approaches include eliciting covariate-specific utilities to quantify risk–benefit trade-offs.

## Eliciting covariate-specific utilities in a phase I-II trials

The first phase I-II design incorporating covariate-specific utility functions was developed to determine the optimal doses, tailored to IMDC risk, among five levels of the novel anti-VEGF tyrosine kinase inhibitor sitravatinib as first-line therapy in patients with mccRCC.^4^ Toxicity was defined as Y_T_ = time to DLT within 3 months, whereas efficacy, Y_E_, was evaluated by measuring objective response by imaging studies performed at 3 months from treatment initiation. Based on the Response Evaluation Criteria in Solid Tumors (RECIST) version 1.1, Y_E_ was defined as an ordinal variable with four levels, progressive disease (PD), stable disease (SD), partial response (PR), or complete response (CR). Prognostic risk was also an ordinal variable representing three subgroups with increasing disease severity: favorable, intermediate, and poor IMDC risk. The utility functions were tailored to each IMDC subgroup because patients with favorable IMDC risk are less willing to accept excess toxicity risk, whereas those with poor IMDC risk are more likely to tolerate a higher risk of toxicity for a higher chance of efficacy.^4^

The subgroup-specific toxicity utility was defined as U_T,s_ = (Y_T_/C)^(σ)^ with α(s) > 0, where Y_T_ is the time to toxicity, up to a maximum follow-up time of C = 84 days (3 months). The exponent α(s) controls how quickly U_T,s_ increases as Y_T_ increases for IMDC subgroup s. To obtain α(s) for each s, clinical experts in mccRCC were asked to answer the following practical questions: (1) what is the maximum utility, U_T,max_, a patient in each IMDC subgroup can obtain if there is no DLT within 84 days? (2) How many days should patients in each IMDC subgroup remain without DLT to obtain half of the maximum utility, U_T,max_ ? The smaller this time is, the more toxicity patients in this subgroup will be willing to accept. For the first question, the answer was U_T,max_ = 140 for all subgroups. Note that the numerical value 140 has no special meaning and is simply an artifact of the process of specifying the utility function. If desired, the numerical utilities may subsequently be divided by 140 to give a utility range of 0 to 1. Such transformations will not change the results of the decision-making. For the second question, the answers were 79, 42, and 28 days for IMDC favorable-, intermediate-, and poor-risk disease, respectively. Using these values, solving the equation U_T,max_· 0.5 = (Y_T_/84)^(s)^ with U_T,max_ = 140 and Y_T_ = 42, gives α = 3.80, 1.00, and 0.63 for IMDC favorable-, intermediate-, and poor-risk disease.^4^

**Figure 4.**
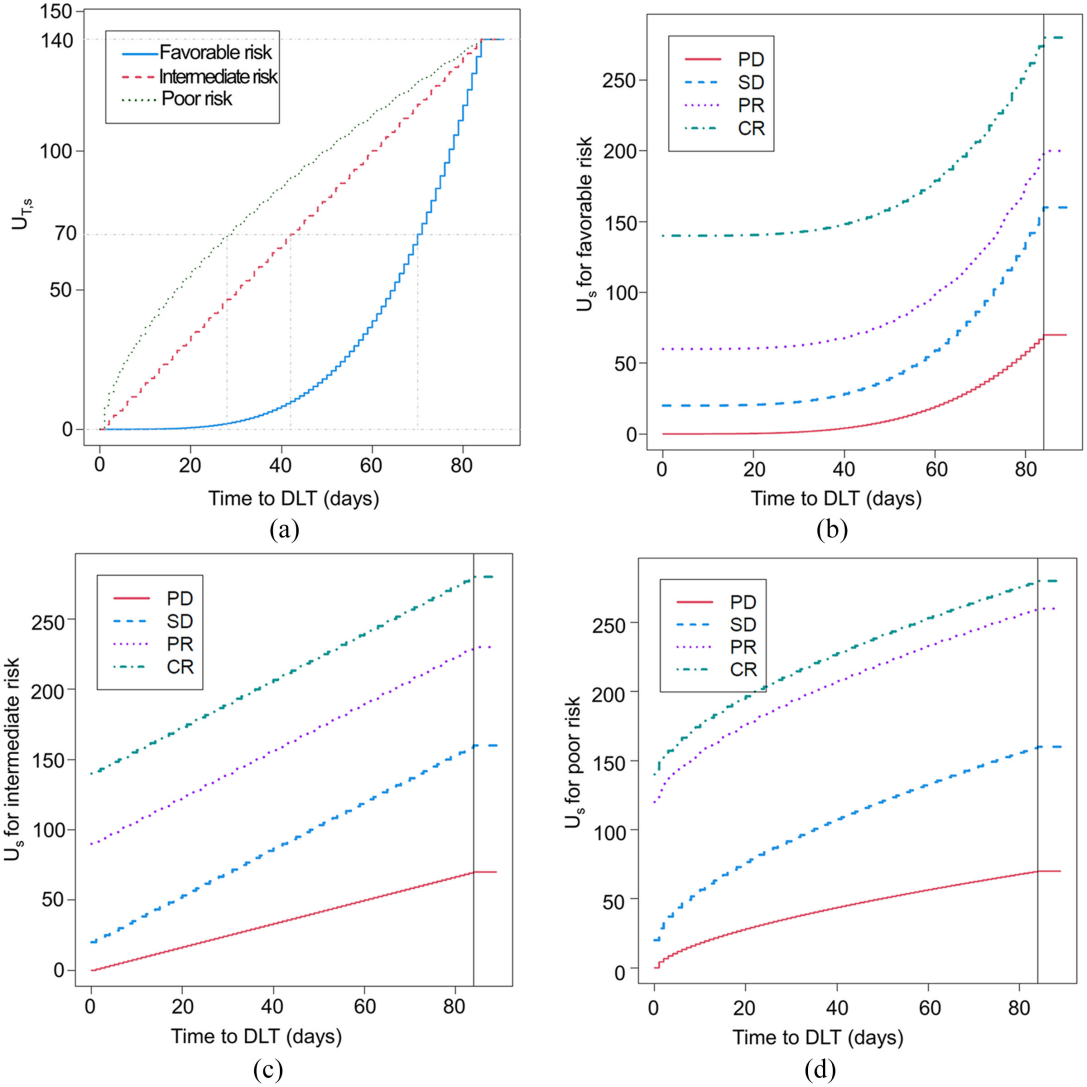
Prognostic covariate-specific utility functions tailored to each of the three IMDC prognostic risk groups of patients with mccRCC. These utility functions quantify the risk–benefit trade-offs to inform dose selection in a phase I-II trial design testing five doses of the targeted agent sitravatinib as first-line therapy in patients with mccRCC. (a) IMDC subgroup-specific toxicity utility functions U_T,s_ encoding that patients with favorable-risk IMDC prognosis are less willing to accept being exposed to DLTs at any time point within 3 months than those with intermediate- or poor-risk disease. (b–d) Total utility U_s_ based on time to DLT and the ordinal efficacy outcome of either progressive disease (PD), stable disease (SD), partial response (PR), or complete response (CR) by imaging at 3 months (84 days) from treatment initiation in patients with IMDC favorable- (b), intermediate- (c), and poor-risk disease (d). Source: adapted from Figure 1 of Lee et al.^4^

To obtain IMDC subgroup-specific utilities, U_E,s_, for the ordinal efficacy outcome, the following questions were asked: (1) How much do we penalize for the occurrence of PD? (2) How much reward do we assign for SD, PR, and CR? For the first question, the answer was to reduce U_T,s_ by half when there was a PD event. The rationale was that PD would offset the benefit obtained by the time without DLT, but patients with PD who experienced DLT later would obtain more benefit than those who quickly developed severe adverse events. For the second question, a CR would give U_E,s_ = 140 and SD would give U_E,s_ = 20 regardless of IMDC subgroup. This is because CR is highly valuable to patients regardless of IMDC subgroup, whereas SD adds a small utility. For PR, U_E,s_ was deemed to be 60, 90, and 120 for IMDC favorable-, intermediate-, and poor-risk disease. This reflects the higher importance of achieving PR in aggressive poor-risk mccRCC. The total utility of (Y_T_, Y_E_) was defined as follows: let U_s_ = U_T,s_/2 if Y_E_ is PD. Otherwise, let U_s_ = U_T,s_ + U_E,s_. The derived utility functions are shown in Figure 4.^4^ While the U_s_ for an outcome with CR and no DLT during the follow-up period is 280 for all subgroups, how the utilities of intermediate outcomes vary is different for each subgroup, as shown in the figure.

The phase I-II design was based on a Bayesian hierarchical model for regression of Y_T_ and Y_E_ on dose d and subgroup s. A joint regression model for [Y_T_, Y_E_—d, s] was assumed with additive dose and subgroup effects for each outcome (Figure 1(a)), using the IMDC prognostic subgroups identified in earlier research^20,22,23^ and thus avoiding the problem of determining new prognostic subgroups from baseline covariates. Such a task would have been very difficult to accomplish dependably due to the limited sample size and sequentially adaptive decision-making used in phase I-II trials. The design efficiently borrowed information across subgroups and sitravatinib doses through the hierarchical structure and also adaptively clustered adjacent ordinal subgroups having similar effects for improved subgroup-specific treatment decision-making. For detailed mathematical construction, refer to equations (1)–(5) in Lee et al.^4^

As a safety constraint, the design specified that an untried dose may not be skipped in any of the subgroups. In order for a dose to be selected and used to treat the next patient in each subgroup, it also had to satisfy two acceptability criteria based on a specified fixed upper limit π*_PD,s_ for the probability π_PD_(d,s) of PD at 3 months and a specified fixed subgroup-specific upper limit π*_DLT,s_ for the probability π_DLT_(d,s) of DLT within 3 months. These safety and efficacy acceptability rules were applied after at least 20 patients in total were fully followed for up to 3 months. A dose d was unacceptable for subgroup s if

(5) $$\mathrm{Pr}\left[\pi_{\mathrm{PD}}\left(d,s\right)>\pi {*}_{\mathrm{PD},s}|\mathrm{data}\right]>.85\;\mathrm{or}\;\mathrm{Pr}\left[\pi_{\mathrm{DLT}}\left(d,s\right)>\pi {*}_{\mathrm{DLT},s}|\mathrm{data}\right]>.85$$

Based on historical data,^38^π*_PD,s_ values .20, .35, and .35 were used for the favorable-, intermediate-, and poor-risk subgroups, respectively. Furthermore, π_DLT_(d,s) > .40 was considered unacceptable for any subgroup, so π*_DLT,s_ = .40 for all s. Patients in each subgroup were adaptively randomized in a way that tends to select acceptable but less explored doses. In each subgroup, the dose chosen for a given patient could be above, below, or the same as that of the previous patient in that subgroup because the design does dose-finding and not dose escalation. Simulations showed that the design compared very favorably to a one-size-fits-all phase I-II design that ignored prognostic subgroups and a design that ran a separate phase I-II trial for each subgroup. The use of subgroup-specific utility functions tailored to the IMDC covariate informed more accurate dose-finding decisions.^4^ Future designs may be developed to allow for more complex outcomes, such as time-to-event survival endpoints.

## Conclusion

Because contradictory conclusions can arise depending on which outcome scale is used, it is necessary to use an estimand that reflects the goals and values of individual patients and other stakeholders in dose-finding studies. The use of utilities allows the explicit incorporation of risk–benefit trade-offs in phase I-II clinical trials. Utilities also may be tailored to specific patient covariates, such as prognostic risk subgroups, to facilitate better informed patient-specific decisions. Rigorous utility elicitation can be facilitated by advances in psychometry, visual aids, and interactive software tools.
